# *ROS1*-positive non-small cell lung cancer: from genomics to treatment decisions

**DOI:** 10.3389/fonc.2026.1739598

**Published:** 2026-02-02

**Authors:** Mylène Wespiser, Romane Gille, Maurice Pérol

**Affiliations:** Department of Medical Oncology, Centre Léon Bérard, Lyon, France

**Keywords:** clinical trial, drug resistance, non-small cell lung cancer, *ROS1* rearrangement, tyrosine kinase inhibitor, oncogene-addicted

## Abstract

*ROS1* rearrangements define a distinct, targetable subset of non–small cell lung cancer (NSCLC), representing ~2% of non-squamous cases and frequently presenting with metastatic disease and CNS involvement. Multiple ROS1 tyrosine kinase inhibitors (TKIs)—from crizotinib to newer agents such as entrectinib, lorlatinib, repotrectinib, taletrectinib, and the highly selective zidesamtinib—have improved systemic and intracranial outcomes, although resistance remains inevitable and biologically diverse, involving both on-target kinase mutations and off-target mechanisms. This review synthesizes current knowledge on ROS1 biology, diagnostic strategies, therapeutic options, and resistance mechanisms. We outline ROS1 fusion architecture and signaling, highlight partner-specific features, and summarize available diagnostic modalities. In clinical practice, RNA-based next-generation sequencing (NGS), often preceded by immunohistochemistry screening, provides the most sensitive approach for fusion detection and resistance profiling. Given the expanding therapeutic landscape and increasing complexity of treatment sequencing, we adopt a pragmatic, practice-oriented framework. CNS-penetrant next-generation TKIs with activity against common resistance mutations now constitute preferred first-line therapy. Repotrectinib and taletrectinib show strong systemic and intracranial efficacy, including activity against *ROS1 G2032R*, whereas zidesamtinib offers high selectivity with encouraging early data. Pemetrexed-based chemotherapy remains an effective option, whereas immune checkpoint inhibitors provide limited benefit. At progression, molecular reassessment is essential to guide tailored therapy. Looking ahead, priorities include optimizing sequencing strategies, evaluating perioperative targeted approaches, and incorporating genomic monitoring to anticipate resistance. These advances are reshaping the natural history of *ROS1*-rearranged NSCLC and supporting a more durable, precision-driven treatment paradigm.

## 1 Introduction

Lung cancer remains the leading cause of cancer-related mortality worldwide, accounting for more than 1.8 million deaths each year (1). Non–small cell lung cancer (NSCLC) represents approximately 85% of all lung cancer cases and encompasses a heterogeneous group of tumors with distinct molecular and histologic characteristics. Over the past two decades, the identification of oncogenic driver alterations, most notably *EGFR* mutations and *ALK* rearrangements, has profoundly transformed the therapeutic landscape of advanced NSCLC, establishing molecularly targeted therapy as a new paradigm in precision oncology. Recently discovered, *ROS1* rearrangements have defined a distinct molecular subset of NSCLC since 2007 (2, 3). It occurs in approximately 2% of NSCLC, corresponding to an estimated 50,000 new cases diagnosed each year worldwide (1). *ROS1* rearrangements result in the constitutive activation of the ROS1 tyrosine kinase domain, driving oncogenic signaling and tumor proliferation. Although rare co-occurrences with EGFR or KRAS mutations have been reported, ROS1 fusions are mutually exclusive to most other oncogenic drivers (4).

Over the past decade, several generations of ROS1-targeted tyrosine kinase inhibitors (TKIs), from crizotinib to newer agents such as lorlatinib, repotrectinib, taletrectinib, and zidesamtinib, have demonstrated both strong systemic and intrecranial efficacy, leading to substantial and durable responses. In this review, we aim to provide a comprehensive and up-to-date overview of the current knowledge regarding the management of *ROS1*-positive NSCLC as of 2025. We will discuss recent advances in our understanding of *ROS1* tumor biology, the diagnostic modalities to detect *ROS1* rearrangements, and how to integrate the therapeutic progress achieved through the successive generations of ROS1-targeted TKI into the treatment strategy. As treatment options continue to expand across both early-stage and metastatic disease, as well as the knowledge about resistance mechanisms, optimizing the sequencing of therapies has become increasingly relevant. This review aims to offer clinicians a broad, precise, and practically useful synthesis of the latest data while also outlining emerging directions and future challenges in *ROS1*-rearranged NSCLC.

## 2 *ROS1* gene and fusions

The *ROS1* proto-oncogene, discovered in the 1980s, resides on chromosome 6q22.1 and encodes a 2347-amino acid receptor tyrosine kinase that belongs to the insulin receptor family (5, 6). The ROS1 protein is organized as a single transmembrane pass, with a large extracellular N-terminus (exons 1–34) containing β-propeller domains and fibronectin type III repeats. It is followed by a juxtamembrane segment, a tyrosine kinase domain (exons 36–42 in the canonical isoform), and a C-terminal tail (7). Interestingly, phylogenetic analyses have demonstrated significant homology between *ROS1* and *ALK*, sharing more than 80% sequence identity, particularly within the kinase domain (8, 9).

To date, no physiological ligand for ROS1 has been identified in humans and is still considered an orphan receptor. However, recent studies in mice have suggested potential physiologic roles, including the identification of neural epidermal growth factor–like 2 (NELL2) as a binding partner for murine ROS1 receptor (10), as well as a potential role for extracellular matrix adhesion in ROS1 activation (11). During embryologic development, wild-type ROS1 expression in mice and chicken is needed, especially for kidney collecting duct and intestine but it also persists into multiple adult tissues such as the kidney, gastrointestinal tract, cerebellum, lung, testis, thymus, and bursa (12). Experimentation showed that *ROS1*-knockout mice appear viable and phenotypically normal, aside from male infertility due to epididymal defects (10, 13).

The oncogenic potential of *ROS1* arises from chromosomal rearrangements that fuse its 3′ tyrosine kinase domain of ROS1 to the 5′ region of partner genes. This mechanism was first uncovered in 1986 through DNA transfer from the MCF-7 human breast carcinoma cell line into NIH-3T3 fibroblasts, revealing homology to the avian v-ros oncogene (14). One year later the same team identified the *FIG* (also known as *GOPC*)–*ROS1* fusion in a glioblastoma cell line, establishing the oncogenic role of ROS1 rearrangements (15). In 2007, a study of phosphotyrosine signaling profiles across 41-NSCLC using a phosphoproteomics approach identified *ROS1* fusions in a separate subset, paving the way for their clinical recognition and pharmacological development (2).

**Figure 1:**
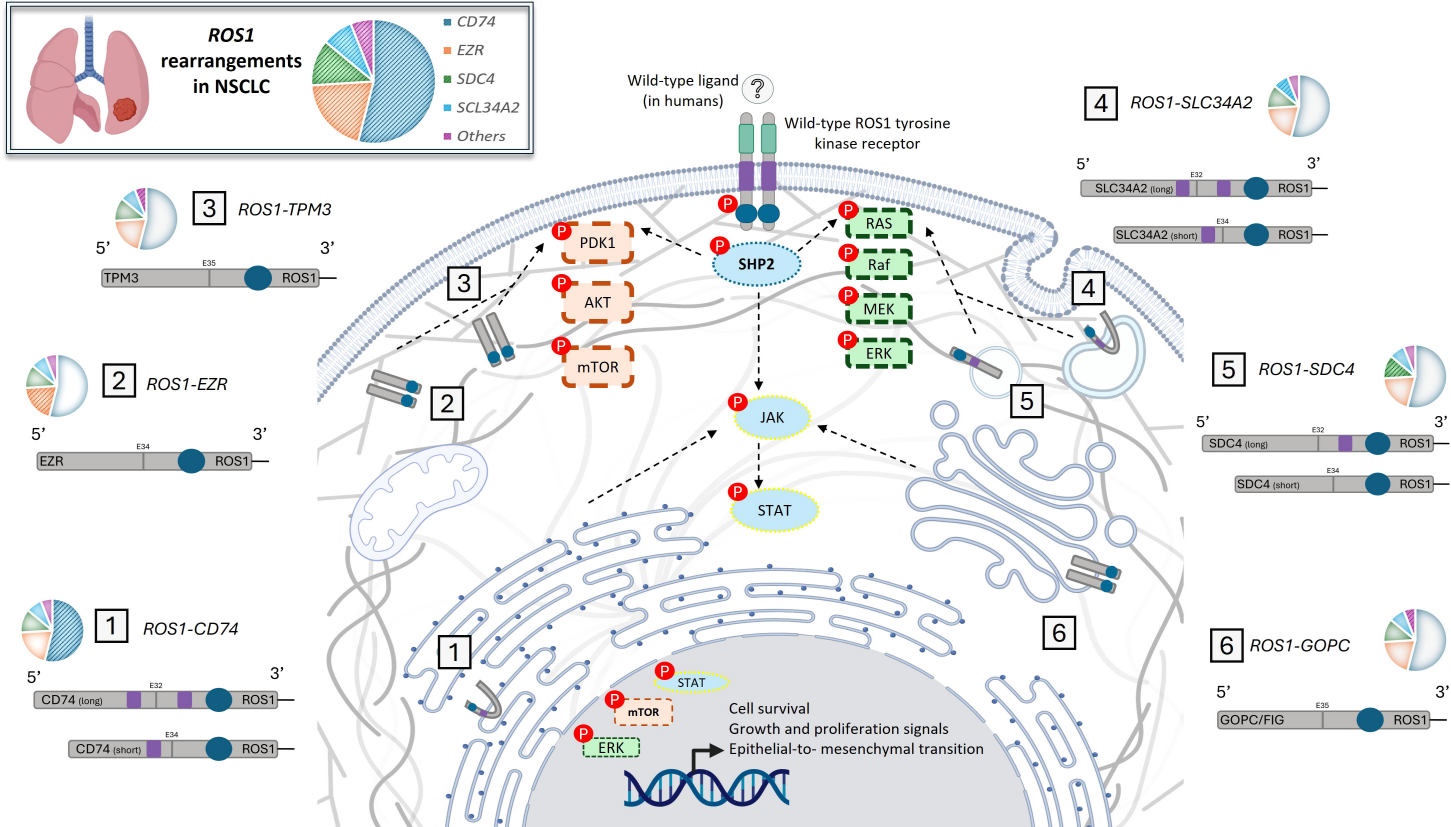
Schematic representation of oncogenic ROS1 fusion proteins and downstream signaling pathways in *ROS1* rearranged NSCLC. *ROS1* rearrangements generate constitutively active chimeric tyrosine kinases in non–small cell lung cancer (NSCLC), in which the ROS1 kinase domain (3′ end) fuses with the 5′ partner gene. The most frequent fusion partners include *CD74*, *EZR*, *SDC4*, *SLC34A2*, *TPM3*, and *GOPC/FIG*, each conferring distinct subcellular localization and signaling properties. In the absence of an identified physiological ligand in humans, these fusions drive ligand-independent activation of ROS1 and its downstream signaling cascades, notably the SHP2–RAS–RAF–MEK–ERK, PI3K–AKT–mTOR, and JAK–STAT pathways. These pathways promote tumor cell proliferation, survival, and epithelial–mesenchymal transition (EMT). Pie charts illustrate the relative prevalence of individual fusion partners across reported NSCLC cohorts.

In cancers, the occurrence of inter- or intra-chromosomal rearrangements may result in *ROS1*-dependent carcinogenesis, leading to the formation of a constitutionally active fusion protein. Mechanistically, this uncontrolled kinase activity and autophosphorylation stimulates multiple canonical pathways, such as MAPK, PI3K/AKT/mTOR, and STAT3. These events promote tumor cell proliferation, survival, and metastasis (16). In NSCLC, breakpoints typically cluster around exons 32-34, whereas the preserved kinase domain spans exons 36- 42. Intrachromosomal events (e.g., 6q22 microdeletions/inversions) are well described in glioblastoma, whereas interchromosomal translocations predominate in NSCLC (9). To date, about 30–50 ROS1 fusion partners have been identified across tumor types. In NSCLC, the most prevalent fusion partners are *CD74* (~44–64%), *EZR* (~14–29%), *SDC4* (~6–16%), *SLC34A2* (~5–12%) and others rarer (*TPM3, ZCCHC8, SLC6A17, GOPC, CCDC6, CTNND2, LRIG3,LIMA1, MSN*,…) (4, 9, 17, 18). Other ROS1 fusion partners have also been described in non-pulmonary malignancies, highlighting the broader oncogenic role of ROS1 across different tumor types (4, 17). The oncogenicity of these fusions depends on the preservation of the ROS1 kinase domain, whereas the 5′ partner determines subcellular localization and downstream signaling (19). *SDC4–ROS1* and *SLC34A2–ROS1* fusions localize to endosomes and preferentially activate the MAPK pathway via SHP2, whereas *CD74–ROS1* localizes to the endoplasmic reticulum and may preferentially activate the JAK/STAT3 pathway (**Figure 1**) (19, 20). These distinct molecular characteristics may be associated with different clinical profiles and drug sensitivity outcomes. For example, *CD74–ROS1* was associated with higher rates of central nervous system metastases in a retrospective study of 36 *ROS1*-positive NSCLC tumors (21).

## 3 Diagnosis of *ROS1* fusions

The detection of *ROS1* rearrangements in clinical practice relies on complementary diagnostic approaches, each with specific advantages and limitations. It is mandatory at the metastatic stage in non-squamous NSCLC and for non-smokers in squamous-cell carcinoma. By analogy with *ALK*-positive NSCLC, *ROS1* rearrangement detection should also be recommended in the early stages of NSCLC before making a decision of perioperative treatment, including immunotherapy, or consolidation with durvalumab in locally advanced disease.

Immunohistochemistry (IHC) is often used as an initial screening tool because of its broad availability, low cost, and rapid turnaround time. Commercial antibodies (e.g., D4D6 clone) can detect ROS1 protein expression in formalin-fixed paraffin-embedded (FFPE) tumor samples, but with a lack of specificity. False positives have been reported mainly due to non-specific cytoplasmic staining or background signal observed in normal lung tissues, including alveolar macrophages and type II pneumocytes, particularly in tumors with abundant mucin or necrosis (9, 22). In addition, false-negative results may occur (approximately 3%–5% depending on the series) due to inadequate tissue fixation, heterogeneous or low fusion protein expression, or inadequacy between antibody clones and platforms (16, 23, 24). Therefore, IHC alone is insufficient to establish a diagnosis and requires confirmatory genomic testing.

Fluorescence *in situ* hybridization (FISH) with a break-apart probe was for a long time regarded as the gold standard in clinical trials evaluating ROS1-targeted therapies. This single-gene assay uses two labeled fluorescents (red and green), each targeting either the 5’ or 3’ regions of the *ROS1* gene. Standard criteria define positivity as the presence of split signals or atypical patterns, such as isolated 3′ signals, in at least 15% of evaluable tumor nuclei (3, 25). It enables the detection of rearrangements independent of the fusion partner. Therefore, the inability to identify the fusion partner can make this method less sensitive in cases of complex or cryptic rearrangements. Interpretation can also be challenged in samples with low tumor cellularity or mucinous histology. However, FISH remains a reliable and widely validated test for diagnosing *ROS1*-rearranged NSCLC (9).

The reverse transcriptase polymerase chain reaction (RT-PCR) is an alternative and relatively cost-effective method for detecting already known ROS1 fusion RNA transcripts by amplifying their expressed complementary DNA. It enables precise partner identification and structural resolution of the fusion event. Compared with FISH, RT-PCR showed good sensitivity (even in the case of low-level variant allele frequencies) but lower specificity (~85%), as requiring high-quality RNA from well-preserved RNA extracted from FFPE samples. Moreover, commercial RT-PCR tests are designed to detect a panel of the most common fusion partners (e.g., *CD74, SDC4, SLC34A2, GOPC, EZR, TPM3, LRIG3*; AmoyDx RT-PCR assay) and therefore miss novel or rare rearrangements (26).

Next-generation sequencing (NGS) has increasingly emerged as the reference method for comprehensive molecular profiling. DNA-based NGS panels are capable of identifying *ROS1* breakpoints but may fail to detect fusions when intronic breakpoints occur in large or poorly covered regions. In contrast, RNA-based NGS directly demonstrates the expressed fusion transcript, allowing the precise identification of the partner gene with high sensitivity and specificity. Importantly, NGS simultaneously surveys several actionable oncogenic drivers, which is clinically relevant, particularly by providing information on co-mutations and the emergence of resistance mechanisms. This strategy is now often prioritized as a frontline diagnostic tool in centers with access to broad RNA-based NGS panels (23, 27).

Liquid biopsy using plasma NGS of circulating tumor DNA (ctDNA) enables the detection of oncogenic gene fusions, including *ROS1* rearrangements. Although false negatives may occur in patients with low ctDNA shedding, typically those with limited intrathoracic disease or isolated brain metastases, plasma-based assays have demonstrated high specificity and increasing sensitivity for fusion detection in advanced NSCLC. Sensitivity is closely related to tumor DNA allelic fraction. In the LIBELULE trial, early ctDNA profiling identified actionable genomic alterations in approximately 13% of cases where tissue analysis was non-informative, confirming the clinical utility of liquid biopsy as a complementary approach to tissue testing when assessing fusion-driven tumors (28, 29). Furthermore, ctDNA can also be used for longitudinal monitoring of *ROS1* rearrangements throughout the disease course and may identify emerging resistance mechanisms to guide subsequent therapeutic decisions.

**Figure 2:**
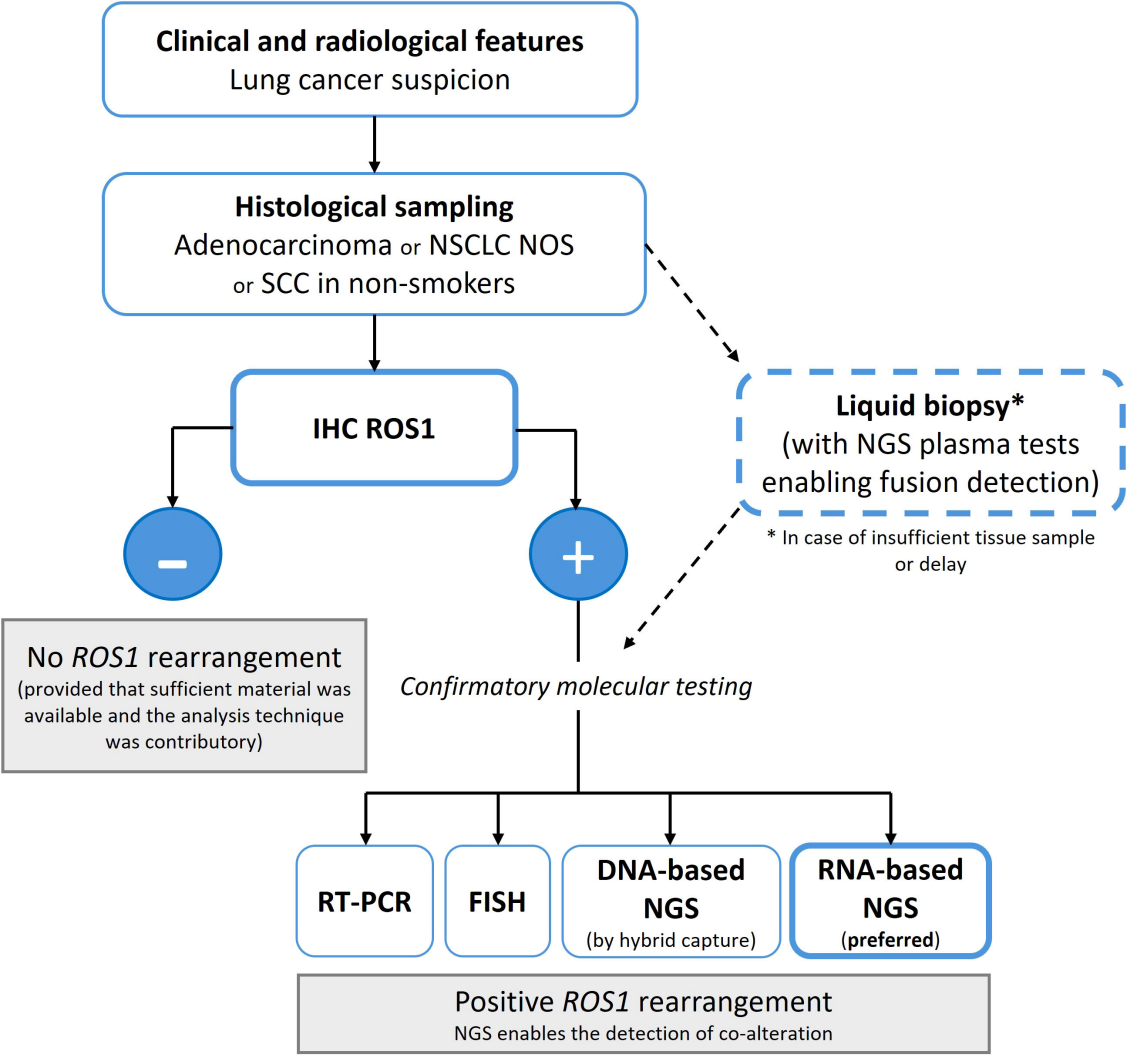
Diagnostic testing algorithm for the detection of *ROS1* rearranged NSCLC. This flowchart summarizes a pragmatic diagnostic approach for identifying *ROS1* rearrangements in non-small cell lung cancer (NSCLC). Immunohistochemistry (IHC) serves as an initial screening tool, with positive cases requiring confirmatory molecular testing by fluorescence *in situ* hybridization (FISH), reverse transcriptase polymerase chain reaction (RT-PCR), or next-generation sequencing (NGS)-preferably RNA-based assays. Liquid biopsy using specific plasma NGS may be considered when tissue is insufficient or unavailable, allowing non-invasive detection of *ROS1* fusions.

Diagnostic workflows are adapted to local resources in daily practice (**Figure 2**). A pragmatic algorithm involves using IHC as an initial screen, followed by confirmation with either NGS (RNA-based preferred) or FISH, depending on the availability of the method. An NGS-first approach is increasingly favored in well-resourced settings, with IHC serving as a triage tool. This layered strategy balances efficiency, cost, and diagnostic precision to ensure the accurate identification of patients with *ROS1*-positive NSCLC who may benefit from targeted therapies. Looking ahead, artificial intelligence has substantial potential to support diagnostic workflows, particularly within molecular biology and pathology, where the analysis of FFPE slides and other histopathological data could be integrated with molecular profiles to enhance diagnostic precision and efficiency (30).

A reliable diagnostic framework is essential because *ROS1*-positive NSCLC with distinctive demographic and histologic features requires tailored therapeutic strategies.

## 4 Clinical and histological presentation

The median age of patients with *ROS1-*positive NSCLC onset is younger (~45–50 years) than that of patients with NSCLC in general. In the reported series, a predominance of females, Asian patients, and never- or light-smokers was observed (3, 31). The predominant histological type is adenocarcinoma, with acinar and solid subtypes being more frequent, but lepidic, papillary, mucinous, signet ring cell subtypes of adenocarcinomas have also been described (32–34). Thyroid transcription factor-1 (TTF1) is expressed in >90% of cases. *ROS1* rearrangements are rarely detected in other histological subtypes, such as squamous cell carcinoma, pleomorphic carcinoma, or large cell carcinoma.

These tumors are characterized by a high metastatic potential and are often diagnosed at stage IV (>80%), which may be linked to the rarity of genomic testing in the early stages. They present a propensity for lymphatic dissemination and a marked neurotropism, as reflected by the high incidence of central nervous system involvement at diagnosis (36-40%) and during evolution, regardless of the fusion partner (35, 36). Miliary patterns of lung metastases have been reported. Several authors have reported an increased thromboembolic risk in patients with *ROS1-*positive NSCLC (37).

## 5 Treatment options

Treatment should be optimized on an individual basis, leveraging the expanding repertoire of ROS1-targeted TKIs alongside established chemotherapy options.

### 5.1 Tyrosine kinase inhibitors targeting ROS1

ROS1-targeted therapies are primarily classified into type I and type II agents based on their binding modalities within the kinase catalytic domain (**Table 1**). Type I inhibitors are ATP-competitive compounds that reversibly bind to the active conformation of the kinase, occupying the ATP-binding site and competing directly with ATP during phosphorylation. In contrast, type II inhibitors interact with the inactive (DFG-out) conformation, extending from the ATP pocket into an adjacent hydrophobic back pocket located behind the gatekeeper residue, thereby stabilizing the inactive state of the kinase. This distinct binding mode may confer a broader resistance-mutation coverage, as type II inhibitors can overcome specific solvent-front and β-sheet mutations (Cβ6), including *ROS1 L2086F*, which mediates resistance to several type I inhibitors (38).

**Table 1:** Type I and II ROS1 inhibitors.

Inhibitor type	ROS1 inhibitor	Structure	Other targets
Type I	Crizotinib	Noncyclic	*ALK, MET*
	Entrectinib	Noncyclic	*NTRK, ALK*
	Brigatinib	Noncyclic	*ALK*
	Ceritinib	Noncyclic	*ALK*
	Lorlatinib	Macrocyclic	*ALK*
	Repotrectinib	Macrocyclic	*ALK, NTRK*
	Taletrectinib	Noncyclic	*NTRK*
	Zidesamtinib	Macrocyclic	highly selective for *ROS1*
Type II	Cabozantinib	Noncyclic	*ALK, MET, AXL, KIT, RET, VEGFR2, FLT3*
	Foritinib	Noncyclic	*ALK*

Type I inhibitors bind to the active conformation of the kinase domain, whereas type II inhibitors recognize the inactive conformation. The table lists the main ROS1 tyrosine kinase inhibitors according to their binding mode, structure (noncyclic or macrocyclic), and known off-target kinases.

ROS1 inhibitors are also classified into successive generations, developed to overcome the *ROS1* resistance mutations to earlier agents and to enhance central nervous system (CNS) penetration.

#### 5.1.1 First-generation inhibitors

Crizotinib was the first approved ROS1-targeted TKI (FDA since 2016), representing a paradigm shift for treating *ROS1*-rearranged NSCLC. In the pivotal PROFILE-1001 trial, crizotinib achieved an overall response rate (ORR) of 72%, a median progression-free survival (PFS) of 19 months, and a median overall survival (OS) of 51 months in TKI-naïve patients (39, 40). These results were subsequently confirmed in multiple independent phase II studies across different geographic cohorts, including the EUCROSS European study (41), the AcSé French trial (42), the East Asia study (43), and the METROS Italian study (44), reporting consistent ORRs ranging from 65% to 80% and a median PFS between 15 and 22 months. Its limitations include poor intracranial penetration and lack of activity against resistance mutations, such as *ROS1 G2032R*. Common toxicities include visual disturbances, gastrointestinal symptoms, peripheral subcutaneous edema, and hepatotoxicity (see **Supplementary Table 1**). Crizotinib remains widely available but has been largely superseded by newer-generation TKIs with improved efficacy and intracranial activity.

Entrectinib is a first-generation multitarget TKI (ROS1, ALK, TRK) with improved brain penetration compared with crizotinib. In an integrated analysis of three phase I–II trials (STARTRK-1, STARTRK-2, ALKA-372-001), entrectinib demonstrated an ORR of 68%, a median PFS of 15.7 months, and a median OS of 47.8 months in TKI-naïve *ROS1*-positive NSCLC patients, with an intracranial ORR (IC-ORR) of 55% (45). However, entrectinib is ineffective against common resistance mutations such as *ROS1 G2032R*, *D2033N*, *L2026M*, and shows limited activity post-crizotinib. Toxicities include fatigue, dizziness, weight gain, dysgeusia, and gastrointestinal effects (see **Supplementary Table 1**) with rare but serious risks of congestive heart failure and QTc prolongation. Entrectinib was approved by the FDA and EMA in 2019 and 2020, respectively.

#### 5.1.2 Second-generation inhibitors

Lorlatinib is a second-generation type I ATP-competitive inhibitor with dual activity against ROS1 and ALK. It is also based on a macrocyclic chemical structure that ensures high brain penetration. In a multicenter phase I/II study, lorlatinib achieved an ORR of 62% and a median PFS of 21.1 months in TKI-naïve patients (n=69), with an IC-ORR of 64% (46). In previously treated patients (n=40), the efficacy was more modest (ORR 35%; median PFS 8.5 months; IC-ORR 50%) (46). Recently, the phase II IFCT-2003 ALBATROS study evaluated the efficacy of lorlatinib after failure of a first-line ROS1 TKI, mainly crizotinib (Duruisseaux et al. *ESMO congress* 2025, 1987MO). Lorlatinib demonstrated a confirmed ORR of 34% (BICR-assessed), a median PFS of 7.4 months, and a median duration of response (DOR) of 20.4 months. Among patients with measurable brain metastases, the IC-ORR reached 92% (n=12/13). Lorlatinib retains no activity against *ROS1 G2032R* on-target resistance mutations emerging after prior ROS1 TKI (47). Its toxicity profile includes hyperlipidemia (72% hypercholesterolemia, 66% hypertriglyceridemia), 42% neuropsychologic-related adverse effects (cognitive changes, mood alterations, speech disturbances), peripheral edema, and weight gain (44%), which may limit long-term tolerability in some patients. Although the FDA did not approve lorlatinib for *ROS1*-positive NSCLC, it has been included in the NCCN and ESMO guidelines since 2019.

Foritinib (SAF-189s), is a type II dual ROS1/ALK kinase inhibitor that was recently evaluated in the phase II SAF-189s-ROS1–001 study conducted in China (48). Foritinib demonstrated strong systemic activity in TKI-naïve patients (n=56), achieving an ORR of 88% (95% CI 76–95) and a median PFS of 22.1 months, with an IC-ORR of 90% in patients with measurable brain metastases (n=19/21). The ORR was 40% (95% CI 21–61) in patients previously treated with ROS1 TKIs. The safety profile was favorable, with hyperglycemia (12% grade 3) and QTc prolongation (6% grade 3) being the most common treatment-related adverse events; no neurotoxicity or sensory disturbances were reported. Foritinib is still under clinical evaluation (SAF001, NCT04237805) and has not yet obtained regulatory approval from the authorities.

Ceritinib, another second-generation type I ATP-competitive inhibitor with ALK and ROS1 activity, demonstrates superior brain penetration compared with crizotinib. In a phase II study (49), ceritinib showed an ORR of 62% and a median PFS of 9 months in mostly crizotinib-naïve patients (n=32). Gastrointestinal adverse events (nausea, diarrhea, vomiting) and hepatotoxicity dominate the toxicity profile, often requiring dose adjustments. ECG monitoring is necessary due to potential QTc prolongation. However, its clinical development in this setting has not led to regulatory approval.

Brigatinib is a second-generation type I ATP-competitive inhibitor with dual ALK/ROS1 activity and enhanced brain penetration. In the phase II BAROSSA study (50), brigatinib demonstrated an ORR of 71% and a median PFS of 12 months in mostly TKI-naïve patients, though data in the post-TKI setting remains limited. Brigatinib exhibits poor activity against the *ROS1 G2032R* resistance mutation. Its safety profile includes gastrointestinal symptoms, hypertension, increased creatine phosphokinase, and pulmonary adverse events (notably early-onset pneumonitis). Brigatinib is not FDA-approved for *ROS1-*positive NSCLC.

Cabozantinib is a type II, non-ATP competitive multikinase inhibitor targeting ROS1, ALK, MET, AXL, KIT, RET, and VEGFR. Preclinical studies suggest activity against selected *ROS1* resistance mutations (e.g. *ROS1 D2033N, L2086F, G2032R*) (51). In addition to preclinical data, isolated case reports have reported responses to cabozantinib, alone or combined with other ROS1 TKIs, in patients harboring the *ROS1 L2086F* resistance mutation, with response lasting approximately 7–11 months (52–54). Nevertheless, these reports are subject to publication bias and do not provide sufficient clinical evidence to support cabozantinib in this setting. Its toxicity profile includes diarrhea, hypertension, palmar-plantar erythrodysesthesia, and cardiovascular events, consistent with other VEGFR-targeting multikinase inhibitors.

Unecritinib (TQ-B3101) is a type I ATP-competitive multikinase inhibitor developed only in China targeting ROS1, ALK, and c-MET. In phase II (NCT03972189), 111 ROS1 inhibitor-naïve patients with advanced or metastatic *ROS1*-positive NSCLC received 300mg twice daily. The ORR was 81%, and the median PFS was 17.2 months. Unecritinib cannot overcome *ROS1* resistance mutations. Among 33 patients with baseline brain metastases, IC-ORR was 72.7%. Grade ≥ 3 treatment-related adverse events (TRAEs) occurred in 51.3% of patients, mainly elevated liver enzyme, whereas ocular and neurologic adverse events were mostly grade 1–2 (55, 56).

Repotrectinib is a macrocyclic, type I ATP-competitive inhibitor designed to target ROS1, ALK, and NTRK fusions, with high CNS penetration and activity against the *ROS1 G2032R* mutation. In the TRIDENT-1 trial, repotrectinib achieved a confirmed ORR (cORR) of 79% in TKI-naïve patients (n=71), an IC-ORR of 89% (n=8/9), and a median PFS of 31.1 (95%CI, 21.9–NE) months. With a median follow-up of 44.6 months, the OS rate was 56% at 4 years (57). In previously treated patients (n=56), the cORR was 41%, median PFS was 8.6 (95% CI, 5.5-14.5) months, and median OS was 25.1 (95% CI, 12.8-32.1) months (57). Repotrectinib was active against *ROS1 G2032R* with an ORR of 59% (n=10/17). The most common adverse events include neurological effects: dizziness (58%, 3% grade ≥3), paresthesia (30%), ataxia (20%), muscular weakness (14%), memory impairment (13%); gastrointestinal disturbance: dysgeusia (50%), constipation (26%), nausea (12%) weight increase (12%) and dyspnea (8%, <1% grade ≥3). Increased AST (aspartate transaminase) and ALT (alanine transaminase) were reported in 18% (1% grade ≥3) and anemia in 26% of cases (58). Repotrectinib has been FDA and EMA-approved since 2023 and 2024 respectively.

Taletrectinib (DS-6051b/AB-106) is a second-generation, CNS-penetrant, type I ATP-competitive inhibitor targeting ROS1 and NTRK but sparing TRKB. It has demonstrated robust activity against several resistance mutations, including *ROS1 G2032R, L2026M, L1951R*, and *S1986F*. In the pooled TRUST-I and TRUST-II analyses (59), taletrectinib achieved high efficacy in TKI-naïve patients (n=160), with a cORR of 88.8%, an IC-cORR of 76.5%, a median PFS of 45.6 months, and a median duration of response (DOR) of 44.2 months. In the post-TKI (mainly crizotinib) setting (n=113), the cORR was 55.8%, the IC-cORR 65.6% with a median PFS of 9.7 months, and a median DOR of 16.6 months. Activity against *ROS1 G2032R*-positive disease was confirmed with a cORR of 61.5% (n=8/13). The toxicity profile is characterized mainly by gastrointestinal adverse events (88%), transaminases elevation (AST 70.2%, ALT 66.5%), and rare neurologic events such as dizziness (16%) and dysgeusia (15%), most of which being grade 1. Treatment discontinuations occurred in 6.5% of patients. Taletrectinib was approved by the FDA in 2025.

#### 5.1.3 Third-generation inhibitors

Zidesamtinib (NVL-520) is a third-generation, macrocyclic, type I ATP-competitive inhibitor specifically designed for high selectivity against ROS1. Kinome profiling demonstrated potent inhibition of ROS1, with minimal off-target activity across 335 kinases; only ALK (2-fold weaker IC_50_) and five kinases (LTK, FAK, PYK2, TRKB, and FER) were inhibited within 10–50-fold of the ROS1 IC_50_, underscoring its high degree of selectivity (60). This selectivity reduces off-target toxicities commonly observed with earlier multitarget TKIs while preserving potent activity against *ROS1*-driven tumors. Its macrocyclic structure further contributes to conformational rigidity, pharmacokinetic stability, and intracranial penetration while also circumventing steric hindrance caused by solvent-front mutations such as *ROS1 G2032R*.

In the phase I/II ARROS-1 trial (NCT05118789), zidesamtinib demonstrated promising efficacy in TKI-naïve (61) and heavily pretreated patients (62). Among 35 TKI-naïve response-evaluable patients, the ORR reached 89%, including 9% complete responses, with a DOR of 96% at 12 months. Among six patients with measurable intracranial disease, the IC-ORR was 83%, including 67% complete responses, and no CNS progression events were observed during follow-up. In TKI-pretreated or post-chemotherapy cohorts (n=117), the ORR was 44%, with PFS of 48% at 12 months and 40% at 18 months. In patients who had received only one prior TKI, ORR reached 51%, with an estimated PFS of 68% at 12 and 18 months. Fifty-three percent of the patients had measurable intracranial metastases at enrollment. Activity against intracranial disease was notable, with an IC-ORR of 85% (n=11/13) in patients pre-treated only with crizotinib (± chemotherapy) and an IC-ORR of 48% (n=27/56) in patients with ≥2 prior ROS1 TKIs, including lorlatinib and/or repotrectinib. Importantly, zidesamtinib demonstrated potent activity against *ROS1 G2032R*, with an ORR of 54% (n=14/26) in patients previously treated with any ROS1 TKI, including lorlatinib and/or repotrectinib. The safety profile was favorable, with the most common TRAEs being peripheral edema (36% any, 0.7% grade 3), constipation (17% any), increased CPK (16% any), dyspnea (15%, 3% grade 3), and transaminase elevation (11%). Dose reductions due to treatment-emergent adverse events (TEAEs) occurred in 10% of patients, whereas treatment discontinuations were infrequent (2%), primarily related to pneumonia (n=3). Zidesamtinib is currently under FDA Real-Time Oncology Review for potential approval.

The various ROS1 TKI differ in dosage, administration schedule, and interactions with food and concomitant medications, as summarized in **Table 2**. Efficacy outcomes in naïve and pretreated patients, including intracranial efficacy and *G2032R* mutation resistance coverage, are outlined in **Table 3**.

**Table 2:** Pharmacology and practical use of ROS1 inhibitors.

Gen	Inhibitor	Dose and schedule	PK/Interactions	Approval status
3^rd^	Zidesamtinib	100 mg QD ± food step-down 75→50 mg (tabs 100/25 mg)	CYP3A4/2C9, P-gp, BCRP, MATE1	FDA RTOR (ongoing)
2^nd^	Taletrectinib	600 mg QD; fasting >2h step-down 400→200 mg (caps only 200 mg)	CYP3A4; PPI,QTc monitoring	FDA-approved 2025
	Repotrectinib	160 mg QD x14d → 160 mg BID ± food; (q12h) step-down 240→160 mg (tabs only 40 mg)	CYP3A4, CYP2D6	FDA-approved 2023; EMA-approved
	Lorlatinib	100 mg QD ± food step-down 75→50 mg (tabs 100/25 mg)	CYP3A4; lipid monitoring	Guideline-listed (not FDA-approved)
	Foritinib	160 mg QD; ± food step-dows 120→ 80mg	CYP3A4;monitor glucose; QTc monitoring	Under clinical investigation
	Ceritinib	450 mg QD with high-fat meal step-down 300 mg (caps only 150 mg)	CYP3A4/2C9; QTc monitoring	Not approved for ROS1
	Brigatinib	90 mg QD x7d → 180 mg QD ± food step-down 90 mg (tabs 180/90 mg)	CYP3A4/2C8; QTc monitoring	Not approved for ROS1
	Cabozantinib	60 mg QD fasting >2h step-down 40→20 mg (tabs 60/40/20 mg)	CYP3A4; QTc monitoring	Not approved for ROS1
1^st^	Entrectinib	600 mg QD ± food step-down 400→200 mg (caps only 200mg)	CYP3A4; QTc monitoring	FDA-approved 2019; EMA-approved
	Crizotinib	250 mg BID ± food (q12h) step-down 200 mg BID → 200 mg QD	CYP3A4	FDA-approved 2016; EMA-approved

**Table 3:** Efficacy of ROS1 TKIs in TKI-naïve (A) and TKI-pretreated (B) *ROS1*-positive NSCLC.

A.									
Drug	Crizotinib	Entrectinib	Ceritinib	Brigatinib	Lorlatinib	Foritinib	Repotrectinib	Taletrectinib	Zidesamtinib
(reference)	Phase 1 (39)	Phase 1-2 (45)	Phase 2 (49)	Phase 2 (50)	Phase 1-2 (46)	Phase 2b (48)	Phase 1-2 (57)	Pooled phase 2 (59)	Phase 1-2 (61)
n	53	168	30	28	21	56	71	160	35
ORR	72%	67.9%	67%	71.4%	62%	88%	79%	88.8%	89%
Median PFS (months)	19.3	15.7	19.3	12.0	21.1	22.1	31.1	45.6	NA
IC ORR	NA	52.1% (25/48) Measurable and non-measurable	40% (2/5) Measurable and non-measurable	0 (0/3) Measurable	64% (7/11) Measurable and non-measurable	90% (19/21) Measurable and non-measurable	89% (8/9) Measurable	76.5% (13/17) Measurable	83% (5/6) Measurable

**Table:** 

B.										
Drug	Entrectinib	Lorlatinib	Lorlatinib	Lorlatinib	Brigatinib	Foritinib	Repotrectinib	Taletrectinib	Zidesamtinib	
(reference)	Phase 1-2 (45)	Phase 1-2 (46)	Phase 2 (ALBATROS)^¤^	(French RW cohort)^£^	Phase 2 (BAROSSA)	Phase 2b (48)	Phase 1-2 (TRIDENT-1) (57)	Phase 2 (TRUST I-II) (59)	Phase 1-2 (ARROS-1) (61)	
n	18	40	50	80	19	25	56	113	117 any prior TKIs (≥ 2, with L or R, ± CT)	55 1 prior ROS1 TKI (C or E) ± CT
ORR	11.1%	35%	34%*	45%	31.6%	40%	41%	55.8%	44%*	51%*
Median DOR (months)	NA	13.8	20.4	7.4	NA	7.0	17.8	16.6	78% at 12 mo	93% at 12 mo
Median PFS (months)	4.7	8.5	7.4	7.1	7.3	5.5	8.6	9.7	48% at 12 mo	68% at 12 mo
IC-ORR (n)	18.8% (3/16)	50% (12/24)	92.3% (12/13)	72% (33/46)	66.7% (4/6)	40% (6/15)	38% (5/13)	65.6% (21/32)	48% (27/56)	85% (11/13)
*ROS1 G2032R* coverage	no	no	no	no	no	no	Yes ORR 59% (n=10/17)	Yes ORR 61.5% (n=8/13)	Yes ORR 54% (n=14/26)	

It is important to note that, given the heterogeneity of the patients included in these studies, no direct comparison can be made between these different clinical trials.

*BICR-assessed (Blinded independent central review); ¤Duruisseaux et al. ESMO congress 2025. ^£^Girard et al. ESMO congress 2022.

**Figure 3:**
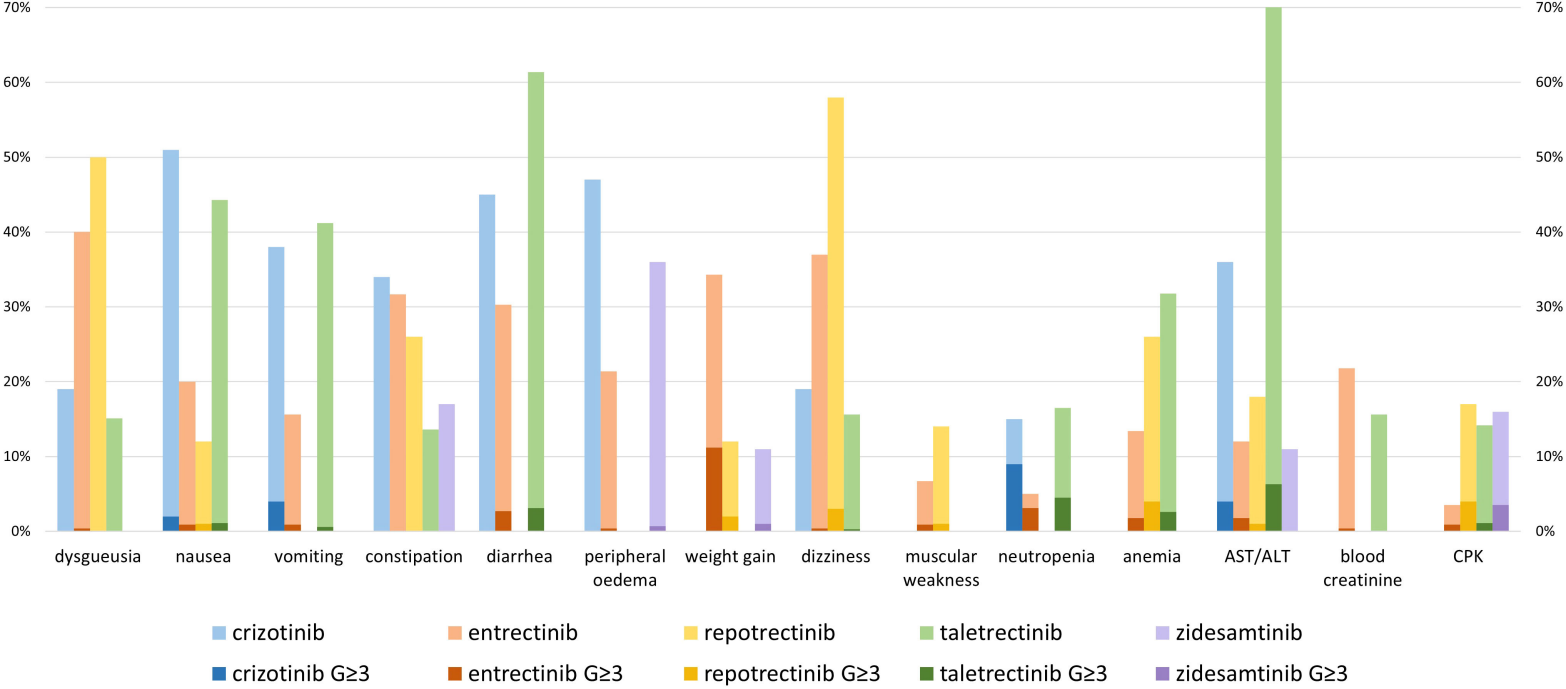
Selected TRAE (occurring in ≥10% patients, all grades) with crizotinib (39), entrectinib (45), repotrectinib (58), taletrectinib (59), and zidesamtinib (61). Light-colored bars indicate the percentage of all-grade toxicities and include dark-colored bars representing the percentage of grade ≥3 toxicities. It is important to note that, given the heterogeneity of the patients included in these studies, no direct comparison can be made between these different clinical trials.

**Figure 3** highlights the most clinically relevant toxicities of crizotinib (n=53) (40), entrectinib (n=224) (45), repotrectinib (n=426) (58), taletrectinib (n=352) (59), and zidesamtinib (n=432) (61) based on the data available to date. TRAEs occurring in ≥10% of patients are detailed in the **Supplementary Table 1**.

### 5.2 Chemotherapy

Before the advent of targeted therapies, the standard first-line treatment for advanced or metastatic *ROS1*-positive NSCLC consisted of platinum-based chemotherapy. Among available cytotoxic regimens, pemetrexed associated to platinum chemotherapy was the most frequently used and appeared to confer superior outcomes, achieving longer PFS (median PFS 6.8 months) and higher ORR (45-60%) than when cisplatin or carboplatin was combined with other agents (gemcitabine, docetaxel) (17, 63). Even compared with other oncogene-addicted NSCLC, patients with *ROS1*-fusion achieved better ORR (57.9%) and PFS (median 7.5 months) with platinum plus pemetrexed chemotherapy (64). A retrospective analysis revealed that long responses to pemetrexed (over a year) were more frequent in the *ALK-* and *ROS1*-positive population (65). Mechanistically, this sensitivity may be partly explained by the low expression level of thymidylate synthase mRNA observed in *ROS1*-positive tumors compared with *ROS1* wild-type cancers (63).

After disease progression or in case of contraindication to pemetrexed, other cytotoxic drugs like taxane, gemcitabine, vinorelbine… may be used, following the same approach as in non–oncogene addicted NSCLC. Regarding bevacizumab, no specific data are available for *ROS1*-positive NSCLC. In different series, some patients received bevacizumab-based regimens, without comparative data to assess its efficacy in this molecular subgroup, and no specific safety signal was reported. The single-arm phase II GFPC 06–2018 study assessing atezolizumab with or without bevacizumab in association with platinum-pemetrexed in *EGFR/ALK/ROS1*-positive NSCLC failed to include *ROS1*-positive NSCLC in the bevacizumab cohort (66). Given the lack of specific data, the same principles applied to non–oncogene addicted NSCLC can guide the use of bevacizumab.

### 5.3 Immunotherapy

Regarding immunotherapy, his use differs from that in non–oncogene-addicted NSCLC. Data on the efficacy of immune checkpoint inhibitors (anti-PD-1/PD-L1) in *ROS1*-positive NSCLC are limited and largely derived from small retrospective series. PD-L1 expression level appears highly variable, with some studies reporting TPS ≥50% in up to 40%–60% of cases, while others describe uniformly low expression. High expression of PD-L1 is not predictive of immunotherapy efficacy, in fact, his upregulation is oncogene-driven rather than a marker of true tumor immunogenicity. A preclinical study demonstrated *in vitro* that blocking the ROS1 fusion with a specific siRNA reduced the expression of both ROS1 and PD-L1 on the surface of HCC78 cells. The authors showed that ROS1 oncogene possesses distinct signaling pathways regulating PD-L1 expression, specifically through the SHP2 and c-Jun pathways (67). Tumor mutational burden is generally low (less than 10 mut/Mb), even lower than other NSCLC, including *EGFR*-mutant NSCLC (68–70).

Clinical activity of anti–PD-1/PD-L1 monotherapy is poor, with ORR ranging from 0% to 20% (68, 69, 71). In the ImmunoTarget study, which retrospectively evaluated the activity of PD-1/PD-L1 inhibitors across oncogene-addicted NSCLC, only 7 patients with *ROS1* rearrangements were included with a single objective response observed, corresponding to an ORR of 17% (72). No specific data on PFS or OS were reported for this subgroup. More recently, a meta-analysis of 86 studies concluded that anti-PD-(L)1 as a single agent has limited efficacy in *HER2/RET/ROS1*-altered NSCLC (71). Overall, the available data highlight that clinical benefit from immune checkpoint inhibitors appears to be rare and inconsistent in *ROS1*-positive NSCLC, supporting the use of targeted therapies or chemotherapy as preferred treatment strategies in this population (73).

Similarly, very limited data are available regarding the role of chemo-immunotherapy combination in *ROS1*-positive NSCLC. Although not formally excluded, these patients were likely underrepresented in first-line advanced NSCLC trials. A few retrospective studies have reported chemo-immunotherapy outcomes in *ROS1*-positive NSCLC, with ORR ranging from 28.6% to 83%, and median PFS varying between 5.8 and 24.3 months, depending on treatment line setting (69, 74). These disparate data do not allow an accurate picture of the contribution of immunotherapy, compared with that of chemotherapy. Given the overall lack of efficacy of immune checkpoint inhibitors in *ROS1*-positive tumors, the use of immunotherapy in combination with chemotherapy in this setting should not be recommended, especially since the toxicity of TKIs may be increased by prior exposure to immunotherapy.

## 6 Resistance mechanisms to ROS1 TKIs

Despite major clinical advances achieved with TKIs targeting ROS1, the development of resistance remains inevitable for most patients. Resistance mechanisms can be broadly divided into on-target (*ROS1*-dependent) alterations, which directly affect the kinase domain and impair inhibitor binding, and off-target (*ROS1*-independent) mechanisms, which reactivate downstream signaling through alternative pathways or induce phenotypic transformation (23, 54).

### 6.1 On-target resistance mechanisms

The most common form of resistance involves secondary point mutations within the ROS1 kinase domain that alter the drug-binding interface. Structural analyses have classified these mutations according to their location and functional impact on the kinase conformation (17, 60).

The solvent-front mutation *ROS1 G2032R* accounts for approximately 35-40% of all resistance events following crizotinib exposure (4, 38). This substitution replaces a small glycine residue with arginine (larger amino acid) at the entrance of the ATP-binding pocket, creating steric hindrance that prevents the TKI from fitting properly into the catalytic pocket (75). The *ROS1 G2032R* variant induces high-level resistance to all first- and second-generation inhibitors, including crizotinib, entrectinib, ceritinib, and lorlatinib (47, 76). Novel generation inhibitors (such as taletrectinib) or macrocyclic inhibitors (such as repotrectinib and zidesamtinib) have been rationally designed to minimize bulk within this region, thereby maintaining activity against this sterically restrictive mutation (23, 60).

The gatekeeper mutation *ROS1 L2026M*, occurring in approximately 5-10% of resistant cases, replaces a small amino acid (leucine) with a larger one (methionine), altering the hydrophobicity and volume of the pocket. This reduces the affinity of TKI without necessarily blocking access completely (54). The gatekeeper mutation acts as a “modified key” within the ATP site, leading to cross-resistance to several TKIs.

Although rarer, there are other clinically relevant mutations, including:

*ROS1 D2033N*, a hinge-binding site mutation, disrupts hydrogen bonding with inhibitors, resulting in intermediate resistance to crizotinib and lorlatinib, but is often sensitive to repotrectinib.

*ROS1 S1986F/Y*, activation loop mutation within the αC-helix, stabilizes the active conformation of the kinase, reducing the efficacy of ATP-competitive type I inhibitors, but certain macrocyclic or compact molecules (such as repotrectinib, talectrectinib, or zidesamtinib) can still bind effectively due to their structural flexibility.

*ROS1 L2086F* or *L2000V* alter the hydrophobic back pocket. *L2086F* introduces a bulky aromatic side chain (phenylalanine), which distorts the geometry of the hydrophobic pocket. As a result, *ROS1 L2086F* confers resistance to all type I ROS1 TKIs (including crizotinib, entrectinib, lorlatinib, repotrectinib, taletrectinib, and zidesamtinib) (18, 47). Type II inhibitors appear to be effective in this condition, based mainly on preclinical data and a few clinical data (54). Similarly, *L2000V*, located nearby in the C-terminal lobe, alters the local hydrophobic environment and may reduce inhibitor affinity.

Overall, these mutations confer intermediate resistance to crizotinib and entrectinib, frequent resistance to lorlatinib, and remain partially sensitive to next-generation inhibitors such as repotrectinib, taletrectinib, and zidesamtinib. Some patients acquire compound or polyclonal mutations, such as *ROS1 G2032R + L2086F* or *L2026M + D2033N*, conferring cross-resistance even to next-generation inhibitors (17).

### 6.2 Off-target resistance mechanisms

When no secondary *ROS1* mutation is identified, resistance is often mediated by bypass pathway activation, which re-establishes downstream signaling independent of ROS1 inhibition. Activation of the EGFR pathway has been observed *in vitro* and in clinical samples as an early adaptive response to ROS1 inhibition, leading to persistent activation of ERK and AKT despite effective ROS1 blockade (54, 75, 77). Similarly, *MET* amplification, *KRAS* mutations, and *MAP2K1* (MEK1) activating mutations have been identified in tumor or plasma NGS analyses from patients treated with entrectinib or lorlatinib (54, 78). In some cases, PI3K–AKT–mTOR pathway reactivation has also been described, particularly in preclinical models of chronic TKI exposure (75, 79).

Beyond signaling reactivation, phenotypic transformations also contribute to TKI resistance. The most frequent is epithelial-to-mesenchymal transition (EMT), characterized by the loss of epithelial markers (E-cadherin) and upregulation of mesenchymal factors, such as vimentin and *ZEB1* (80). This process promotes cellular plasticity, invasiveness, and drug tolerance. Rarely (in about 2% of cases), small-cell lung cancer (SCLC) transformation has been reported in *ROS1*-positive tumors following prolonged TKI exposure, mirroring patterns observed in *EGFR*- and *ALK*-driven disease (81). SCLC transformation during ROS1 TKI therapy is frequently associated with upstream molecular events, such as loss of *RB1* and *TP53* mutations, suggesting a lineage plasticity–driven process.

Thus, these mutational and non-mutational resistance mechanisms highlight the adaptive capacity of tumor cells under targeted therapy pressure. The spectrum of acquired ROS1 resistance mutations varies according to the ROS1 fusion partner, the type and generation of TKI used as first-line therapy, reflecting distinct structural constraints and selective pressures imposed by each inhibitor. Collectively, these findings underscore the polyclonal nature of resistance, where multiple subclones may coexist within the same tumor or between metastatic sites. This intrapatient heterogeneity emphasizes the need for repeated molecular profiling at progression to tailor subsequent lines of therapy.

## 7 Therapeutic strategy in *ROS1*-positive NSCLC

The therapeutic strategy for *ROS1*-positive NSCLC has to integrate available targeted agents, chemotherapy options, and sequencing considerations.

Treatment recommendations should be guided by a multidisciplinary team, taking into account patient age and comorbidities. Patients should be involved in the decision-making process, with particular consideration given to their preferences and the impact of treatment-related adverse effects on quality of life.

### 7.1 Early and locally advanced stages

The management of the early stages of *ROS1*-positive NSCLC generally follows the same principles as non-oncogene-addicted NSCLC, with surgical resection and, when indicated, perioperative chemotherapy.

Recent advances have established the efficacy of perioperative chemo-immunotherapy in resectable NSCLC, demonstrating an improvement in pathological complete response [CheckMate 816 (82), KEYNOTE-671 (83), CheckMate 77T (84)] and OS benefit (CheckMate 816 (82), Keynote 671 (85)). Patients with *ALK* or *EGFR* alterations were excluded from the majority of the phase III trials, and it remains unclear whether *ROS1*-positive NSCLC were included in these studies. The limited efficacy of immune checkpoint inhibitors in *ROS1*-positive NSCLC (as discussed above) does not support the use of perioperative immunotherapy in this setting.

However, early stages of NSCLC dependent on oncogenic addiction may benefit from targeted therapies in an adjuvant setting. The ADAURA study paved the way for *EGFR* mutations by demonstrating that osimertinib given for 3 years after adjuvant chemotherapy, if indicated, provides a benefit in DFS (HR 0.27, 95% CI, 0.21-0.34) and OS (HR 0.49, 95% CI, 0.33-0.73), establishing a new paradigm in this situation. Similarly, after complete resection of *ALK*-positive NSCLC, both alectinib compared with adjuvant chemotherapy (86) and ensartinib given during 2 years after adjuvant chemotherapy compared with a placebo (Yue et al. *ESMO Congress* 2025, LBA66) showed a highly significant DFS benefit (HR 0.24, 95% CI, 0.13-0.45 and HR 0.20, 95% CI, 0.11-0.38, respectively).

To date, no clinical trial has established a benefit of ROS1-targeted therapies in an adjuvant setting. Several studies are ongoing. A phase III study of taletrectinib *versus* placebo as adjuvant therapy is ongoing (TRUST-IV, NCT07154706). A phase II trial evaluating crizotinib in the neoadjuvant setting has been completed, and results are awaited (NCT03088930). The “umbrella” phase II NAUTIKA1 trial (NCT04302025) assesses the efficacy and safety of multiple targeted therapies as perioperative (neoadjuvant and adjuvant) treatment in patients with resectable stage IB–III NSCLC, including *ROS1*-positive NSCLC treated with entrectinib. Preliminary results have been reported only for *ALK*-positive NSCLC; further outcomes are pending (87). There are a few case reports describing patients with resectable *ROS1*-positive NSCLC who received ROS1 TKI in the neoadjuvant setting before surgical resection (88–90). No conclusion can formally be drawn for clinical practice.

In the unresectable locally advanced stage, the treatment approach is similar to that of non–oncogene-addicted NSCLC, consisting of concurrent chemoradiotherapy. In *ROS1*-positive NSCLC, the preferred choice of chemotherapy would be the combination of cisplatin or carboplatin-pemetrexed, given the data available in stage IV disease. For consolidation treatment after chemoradiation, a similar rationale to that used for *EGFR*-mutant tumor may apply. Indeed, osimertinib demonstrated in the LAURA phase III trial a significant improvement in PFS (HR 0.16, 95% CI, 0.10-0.24) in this setting and a trend toward OS benefit, although data remain immature (91). It also significantly improved intracranial PFS (HR 0.17, 95% CI, 0.09-0.32), implying a protective effect against brain relapses (92). These findings could support the role of targeted therapies as consolidation treatment post chemoradiotherapy in oncogene-addicted NSCLC, rather than durvalumab (93), for which no specific data are available in *ROS1*-positive NSCLC. However, to the best of our knowledge, no clinical trial is currently ongoing to specifically address this question.

### 7.2 Stage IV disease

#### 7.2.1 First-line treatment of stage IV ROS1-positive NSCLC

First-line treatment of advanced *ROS1*-positive NSCLC should ideally involve the most effective and best-tolerated TKI currently available. Similar to the situation with *EGFR* mutations or *ALK* rearrangements, the choice of first-line treatment appears crucial in altering the natural history of the disease and improving patient survival. Delaying disease progression by preventing the emergence of resistance mechanisms appears to be more effective than attempting to overcome them. The frequency of brain metastases at baseline and during the disease course warrants the use of a CNS-penetrant TKI, and alternatives to crizotinib should therefore be preferred in this setting. Interestingly, a phase III trial (NCT04603807) compares entrectinib with crizotinib with intracranial PFS as the primary endpoint. The results are not yet available to date.

Furthermore, regulatory approval status is an additional practical consideration, as not all ROS1 inhibitors are available in every country. In the ASCO Guidelines, first-line options mention crizotinib, entrectinib, or repotrectinib; if not available or tolerated, ceritinib or lorlatinib are possible alternatives (94). Updated 2025 ESMO Guidelines offer the options of crizotinib or entrectinib in the first-line setting and repotrectinib as an option (95). The NCCN Guidelines recommended first-line options include crizotinib, entrectinib, repotrectinib, and taletrectinib (96).

The use of new-generation inhibitors more potent against native ROS1 kinase, with high level of CNS penetration appears to be the best current option to improve first-line treatment and try to change the natural history of the disease. Among these, taletrectinib has demonstrated the most favorable efficacy and safety profile (see section 4.1.2); it therefore appears to be the preferred first-line option. A phase III trial is currently ongoing and aims to compare taletrectinib with crizotinib in *ROS1*-positive locally advanced or metastatic NSCLC previously untreated (TRUST-III, NCT06564324). The tolerance profile of repotrectinib, which has fairly similar activity, makes it more difficult to use over the long term, particularly as a first-line treatment. Zidesamtinib has also shown promising preliminary activity in phase I/II ARROS-1, although data remain immature.

#### 7.2.2 Beyond the first-line setting

Beyond the first-line setting, the treatment principles are similar to those for advanced NSCLC with oncogenic addiction. The therapeutic sequence mainly depends on the first-line agent used. Whenever possible, patients should be systematically evaluated for eligibility and offered enrollment in a clinical trial, particularly in later-line settings where the level of evidence remains limited.

Most available data concern post-crizotinib setting. Clear treatment guidelines following the first-line use of next-generation ROS1 TKIs cannot currently be defined due to the lack of long-term data.

The clinical pattern of disease progression should be assessed first. In cases of oligoprogression, definitive local therapies (e.g. stereotactic radiotherapy, surgery and thermoablation) should be considered to allow the continuation of the initial TKI beyond progression.

In cases of systemic disease progression, systematic rebiopsy or liquid biopsy is strongly advised whenever feasible. Identification of resistance mechanisms may enable treatment personalization in the presence of targetable alterations. Objective response rates observed with lorlatinib, entrectinib, repotrectinib, taletrectinib, and zidesamtinib after prior ROS1 TKI therapy, mainly crizotinib, are summarized in **Table 4**. These data remain limited and are derived from relatively small cohorts and should therefore be interpreted with caution. Repotrectinib is currently the preferred option for post-crizotinib treatment according to the ESMO guidelines (95). In the event of CNS progression, switching to a more CNS-penetrant TKI should be prioritized. Preferred options include zidesamtinib, taletrectinib, and repotrectinib. If these options are unavailable, lorlatinib or entrectinib may be considered.

**Table 4:** Efficacy of subsequent ROS1 TKIs after progression on a first-line ROS1 TKI.

Drug (reference)	Lorlatinib (46)	Entrectinib (45)	Repotrectinib (57)	Taletrectinib (59)	Zidesamtinib^§^ (61)
Post-crizotinib	ORR 35% (n=14/40)	ORR 11% (n=2/18)	ORR 39% (n=18/46)	ORR 53% (n=55/103)	ORR 68% (n=19/28)
Post-entrectinib	(n=1/1)		ORR 22% (n=2/9)	ORR 80% (n=8/10)	ORR 33% (n=9/27)
Post-repotrectinib	NA	NA		NA	ORR 47% (n=8/17)
Post-taletrectinib	NA	NA	NA		ORR 43% (n=3/7)

Objective response rates are shown for each treatment according to prior ROS1 TKI exposure. Data are extracted from published studies as indicated. It is important to note that, given the heterogeneity of the patients included in these studies, no direct comparison can be made between these different clinical trials.

^§^ORR refers to patients previously treated with a ROS1 TKI with or without chemotherapy (i.e., one or two prior lines of therapy).

The only data available for disease progression under next-generation ROS1 TKIs come from the preliminary results of the ARROS-1 trial (62). Zidesamtinib showed significant activity after next-generation ROS1 TKIs, whether or not preceded by crizotinib, with an ORR of 38% (95% CI, 26-52) after ≥2 prior ROS1 TKIs ± chemotherapy (**Table 4**). As zidesamtinib is still not widely available, subsequent treatment should consist of platinum-pemetrexed chemotherapy, without immunotherapy. To the best of our knowledge, no prospective studies have demonstrated the benefit of maintaining ROS1 TKI post-progression in addition to chemotherapy.

At all times during patient management, comprehensive supportive care must accompany systemic treatment to optimize patient outcomes and quality of life.

## 8 Conclusion

*ROS1*-rearranged NSCLC constitutes a rare but clinically significant molecular subset, underscoring the importance of systematic detection strategies, ideally based on RNA sequencing. The treatment advances made in *ROS1*-positive disease mirror that of *ALK*-rearranged tumors, although its lower prevalence has limited the availability of large cohorts and randomized clinical trials. First-generation TKIs with mainly crizotinib and then entrectinib established the initial standard of care; however, the emergence of next-generation ROS1 inhibitors with enhanced potency, central nervous system penetration, and activity against crizotinib resistance mechanisms has markedly improved clinical outcomes. These agents now achieve median progression-free survival durations comparable to those reported with second-generation ALK inhibitors, with promising data emerging for novel compounds such as zidesamtinib, leading to expect a substantial improvement in overall survival in advanced disease settings. Despite these advances, TKIs retain inherent limitations similar with those of other oncogene-addicted NSCLCs with target-independent mechanisms of resistance and tumor heterogeneity, explaining that chemotherapy continues to play a complementary role in disease management. As with *ALK*, targeted therapies have reshaped the natural history of *ROS1*-positive NSCLC, with anticipated benefits extending into earlier disease stages. Critical knowledge gaps remain, particularly regarding resistance mechanisms to newer-generation TKIs and the optimal sequencing of available therapeutic strategies.

## Supplementary material

Supplementary Table 1 Most frequent TRAEs (10% or more, all grades) described with crizotinib, entrectinib, repotrectinib, taletrectinib, and zidesamtinib summarizes the most frequent toxicities of crizotinib (39), entrectinib (45), repotrectinib (58), taletrectinib (59), and zidesamtinib (61) based on the data available to date. As the report and classification of AEs may vary from one trial to another, these data are not comparative.

## Glossary

AE: adverse event
AKT: protein kinase B
ALK: anaplastic lymphoma kinase
ALT: alanine transaminase
AST: aspartate transaminase
BCRP: breast cancer resistance protein
BID: twice daily
caps: capsules
CPK: creatine phosphokinase
CYP: cytochrome P450 enzymes
DNA-NGS: DNA-based next-generation sequencing
DOR: duration of response
EMA: European Medicines Agency
EMT: epithelial–mesenchymal transition
ERK: extracellular signal-regulated kinase
ESMO: European Society for Medical Oncology
FDA RTOR: U.S. Food and Drug Administration Real-Time Oncology Review
FISH: fluorescence *in situ* hybridization
FLT3: Fms-like tyrosine kinase 3
Gen: generation of the tyrosine kinase inhibitor (first, second, or third)
IC-ORR: intracranial objective response rate
IHC: immunohistochemistry
JAK: Janus kinase
KIT: KIT proto-oncogene receptor tyrosine kinase
MATE1: multidrug and toxin extrusion protein 1
MEK: mitogen-activated protein kinase kinase
MET: mesenchymal–epithelial transition factor
mDOR: median duration of response
mo: months
mOS: median overall survival
mPFS: median progression-free survival
mTOR: mechanistic target of rapamycin
NA: not available
NCCN: National Comprehensive Cancer Network
NGS: next-generation sequencing
NSCLC: non–small cell lung cancer
NTRK: neurotrophic tyrosine receptor kinase
ORR: objective response rate
PFS: progression-free survival
P-gp: P-glycoprotein
PI3K: phosphatidylinositol 3-kinase
PK: pharmacokinetics
QD: once daily
RET: rearranged during transfection proto-oncogene
RNA-NGS: RNA-based next-generation sequencing
ROS1: c-ros oncogene 1 receptor tyrosine kinase
RT-PCR: reverse transcriptase polymerase chain reaction
RW: real world
SHP2: Src homology phosphatase 2
STAT: signal transducer and activator of transcription
tabs: tablets
TKI: tyrosine kinase inhibitor
Type I/II: binding mode of the inhibitor (Type I: ATP-competitive active conformation
VEGFR2: vascular endothelial growth factor receptor 2.
